# Supplementary material for: Photochemical alteration of organic carbon draining permafrost soils shifts microbial metabolic pathways and stimulates respiration
Source: Nat Commun. 2017 Oct 3;8:772. doi: 10.1038/s41467-017-00759-2 (PMC5626735; doi:10.1038/s41467-017-00759-2)
Supplement: Supplementary file 1 — Supplementary Information [file 41467_2017_759_MOESM1_ESM.pdf]

Description of Supplementary Files

File Name: Supplementary Information
Description: Supplementary Figures, Supplementary Tables and Supplementary References

File Name: Peer Review File
Description:

Supplementary Table 1: Summary of FT-ICR MS data for permafrost and organic layer DOC that was degraded by or resistant to microbes, or degraded by or produced by sunlight.

**Source**	**Category**[a]	**Count**	**MW**[b]	**O/C**[b]	**H/C**[b]	**Tannin-like** (%)[c]
Permafrost	*Degraded by microbes*	415	479	0.34	1.21	10
	Resistant to microbes	866	482	0.53	1.14	32
	Degraded by sunlight	397	511	0.57	1.03	49
	Produced by sunlight	356	448	0.34	1.17	6
Organic layer	*Degraded by microbes*	397	540	0.62	0.90	65
	Resistant to microbes	1438	504	0.54	1.02	34
	Degraded by sunlight	375	566	0.64	0.88	74
	Produced by sunlight	784	460	0.52	1.02	24

[a] Formulas produced by or degraded by sunlight were previously reported[1].

[b] MW = molecular weight, O/C = oxygen to carbon atomic ratio, H/C = hydrogen to carbon atomic ratio.

[c] Formulas were categorized as tannin-like if their composition met the following criteria: ($0.6 \leq O/C \leq 1.2$, $0.5 \leq H/C \leq 1.5$, $AI_{MOD} < 0.67$), where AI_{MOD} is the modified aromaticity index[2].

Supplementary Table 2: Summary of microbial activity results for experimental incubations of light-exposed and dark-control permafrost and organic layer DOC with native microbial communities. Activity is reported as the mean ± 1 SE of duplicate incubation vials, as well as the mean ± 1 SE of experimental triplicates.

**Source**	**Treatment**	**Experimental Replicate**	**O_2 Consumption** (μM)	**CO_2 Production** (μM)	**Microbial Production** (μg C L^{-1} d^{-1})
Permafrost	Dark-Control	1	17 ± <1	9 ± 1	84 ± 1
		2	13 ± <1	17 ± 9	74 ± 2
		3	11 ± <2	8 ± 1	63 ± <1
		Mean ± SE	*13 ± 2*	*11 ± 4*	*74 ± 6*
	Light-Exposed	1	29 ± <1	10 ± 3	71 ± 2
		2	29 ± <1	41 ± <1	93 ± 1
		3	28 ± <1	23 ± <1	69 ± 5
		Mean ± SE	*28 ± <1*	*25 ± 8*	*78 ± 8*
Organic layer	Dark-Control	1	294 ± 3	199 ± 2	165 ± 1
		2	261 ± 2	219 ± 2	161 ± 1
		3	193 ± 3	128 ± 1	10 ± <1
		Mean ± SE	*249 ± 26*	*182 ± 25*	*112 ± 51*
	Light-Exposed	1	181 ± 2	147 ± 2	3 ± <1
		2	190 ± 5	157 ± 1	7 ± <1
		3	177 ± 1	151 ± <1	3 ± <1
		Mean ± SE	*183 ± 4*	*152 ± 3*	*4 ± 1*

Supplementary Table 3: Chemical attributes of water leached from the upper thawed and permafrost layer of three different soil types on the North Slope of Alaska. These soil types (moist acidic tundra, moist non-acidic tundra, and wet sedge tundra) are broadly representative of pan-arctic soils[3]. Error bars represent standard error of mean of experimental replicates. Replicates were not available for the permafrost layer of Toolik moist, acidic tussock tundra. Dissolved organic carbon (DOC) was measured following[4]. Specific ultra-violet absorbance measured following[5]. Data for Imnavait moist acidic tussock tundra were previously reported[6].

Soil Type	Coordinates	Soil Layer	N	pH	Conductivity ($\mu S\ cm^{-1}$)	DOC (μM)	SUVA ($L*mg\text{-}C^{-1}*m^{-1}$)
Imnavait moist acidic tundra	68°36'51.86"N	Active	3	5.7 ± 0.1	37 ± 3	3054 ± 181	2.7 ± 0.1
	149°18'31.87"W	Permafrost	3	5.8 ± 0.1	9 ± 1	998 ± 27	1.2 ± 0.2
Imnavait wet sedge tundra	68°36'33.89"N	Active	2	5.3 ± <0.1	12 ± <1	1644 ± 70	2.7 ± 0.1
	149°18'52.58"W	Permafrost	2	5.5 ± <0.1	15 ± <1	1595 ± 20	2.3 ± 0.1
Toolik moist acidic tundra	68°37'46.97"N	Active	2	5.3 ± 0.1	7 ± <1	973 ± 18	2.1 ± 0.1
	149°34'49.29"W	Permafrost	1	7	30	2356	1.9
Sagavanirktok moist non-acidic tundra	69°26'52.77"N	Active	2	7.3 ± <0.1	100 ± <1	246 ± 20	1.2 ± 0.5
	148°36'42.21"W	Permafrost	2	7.3 ± 0.1	133 ± 3	1550 ± 68	2.9 ± 0.1
Sagavanirktok moist acidic tundra	69°25'28.70"N	Active	2	6.7 ± <0.1	21 ± 6	481 ± 10	3.5 ± 0.1
	148°41'39.05"W	Permafrost	2	7.7 ± <0.1	177 ± <1	2077 ± 29	1.3 ± <0.1

Supplementary Table 4: Average characteristics of DOC formulas detected FT-ICR MS that were degraded by or produced by sunlight across the five soil types described in Supplementary Table 3. Formulas were assigned to compound classes using the following criteria: Tannin-like: $0.6 \leq O/C \leq 1.2$, $0.5 \leq H/C \leq 1.5$, $AI_{MOD} < 0.67$; Lignin-like: $0.1 < O/C < 0.6$, $0.5 \leq H/C \leq 1.7$, $AI_{MOD} < 0.67$; "Other" is the sum of Lipid-like, Carbohydrate-like, Unsaturated Hydrocarbon, Protein-like, and Condensed Aromatic formulas, which were assigned using the following criteria: Lipid-like: $0 < O/C \leq 0.2$, $1.7 < H/C \leq 2.2$; Carbohydrate-like: $0.6 \leq O/C \leq 1.2$, $1.5 < H/C \leq 2.2$; Unsaturated Hydrocarbon: $0.0 < O/C \leq 0.1$, $0.7 < H/C \leq 1.7$; Protein-like: $0.2 < O/C < 0.6$, $1.5 < H/C \leq 2.2$, $N/C \geq 0.05$; Condensed Aromatic: $0.0 < O/C \leq 1.0$, $0.3 \leq H/C \leq 0.7$, $AI_{MOD} \geq 0.67$.

DOM Source	Layer	Category	Mass	O/C	H/C	Lignin	Tannin	Other
Imnavait moist acidic tundra	Active Layer	Photo-degraded	566	0.64	0.88	21	74	5
		Photo-produced	460	0.52	1.02	71	24	5
	Permafrost	Photo-degraded	511	0.57	1.03	50	49	1
		Photo-produced	448	0.34	1.17	67	6	27
Imnavait wet sedge tundra	Active Layer	Photo-degraded	457	0.59	0.88	33	48	19
		Photo-produced	387	0.44	1.20	77	9	14
	Permafrost	Photo-degraded	475	0.55	1.05	36	42	21
		Photo-produced	409	0.47	1.11	71	14	15
Toolik moist acidic tundra	Active Layer	Photo-degraded	477	0.51	1.08	59	36	5
		Photo-produced	392	0.44	1.17	62	18	19
	Permafrost	Photo-degraded	496	0.65	0.87	15	67	18
		Photo-produced	491	0.45	1.21	70	10	20
Sagavanirktok moist non-acidic tundra	Active Layer	Photo-degraded	528	0.59	0.93	44	52	4
		Photo-produced	337	0.52	1.07	68	26	6
	Permafrost	Photo-degraded	436	0.52	0.82	57	32	11
		Photo-produced	404	0.54	1.23	62	30	8
Sagavanirktok moist acidic tundra	Active Layer	Photo-degraded	537	0.63	0.86	22	67	11
		Photo-produced	372	0.50	0.93	70	20	11
	Permafrost	Photo-degraded	449	0.43	1.12	85	11	4
		Photo-produced	431	0.53	1.30	59	30	11
All Sources	Mean	Photo-degraded	493	0.57	0.95	42	48	10
	SE		13	0.02	0.03	7	6	2
	Mean	Photo-produced	413	0.48	1.14	68	19	14
	SE		14	0.02	0.03	2	3	2
Active Layer Only	Mean	Photo-degraded	513	0.59	0.93	36	55	9
	SE		20	0.02	0.04	7	7	3
	Mean	Photo-produced	390	0.49	1.08	70	20	11
	SE		20	0.02	0.05	2	3	3
Permafrost Only	Mean	Photo-degraded	473	0.54	0.98	49	40	11
	SE		14	0.04	0.06	12	9	4
	Mean	Photo-produced	437	0.47	1.20	66	18	16
	SE		16	0.04	0.03	2	5	3

Supplementary Table 5: Bacterial production measurements for whole water, GF/F filtered, and 0.2 µm filtered fractions of Toolik Lake water exposed to natural sunlight or kept in the dark. These results indicate that GF/F filtration (nominal pore size of 0.7 µm) substantially removes bacteria and thus minimizes bacterial activity compared to unfiltered lake water (i.e., always >90% decrease in production, mean = 93 ± 2% SD), and that 12 hours of natural light exposure in the UV-transparent vessels used in our experiments sterilizes all water fractions (i.e., always >99% decrease in production). Together, these results suggest that the 0.45µm filtration we used for organic layer and permafrost DOC effectively removed bacteria and minimized bacterial activity during photo-incubations. Even GF/F filtration alone, or in conjunction with UV exposure during experimental irradiation, minimized contamination and bacterial activity to 1-10% of that measured in the dark, unfiltered control. These same filtering protocols have been used in our research at Toolik Lake since 2007, and the results thus apply to[1,6–14].

Site Name	Depth	Collection Date	Filter Fraction	Experimental Treatment	Bacterial Production
-	m	-	-	-	$\mu g\ C\ L^{-1} d^{-1}$
Toolik Lake	3	27-May-13	whole water	Light-Exposed 12 hours	0.14
Toolik Lake	3	27-May-13	whole water	Dark-Control 12 hours	15.11
Toolik Lake	3	27-May-13	GF/F	Light-Exposed 12 hours	0.01
Toolik Lake	3	27-May-13	GF/F	Dark-Control 12 hours	1.31
Toolik Lake	3	27-May-13	0.2 µm	Light-Exposed 12 hours	0.29
Toolik Lake	3	27-May-13	0.2 µm	Dark-Control 12 hours	0.04
Toolik Lake	16	27-May-13	whole water	Light-Exposed 12 hours	0.08
Toolik Lake	16	27-May-13	whole water	Dark-Control 12 hours	14.87
Toolik Lake	16	27-May-13	GF/F	Light-Exposed 12 hours	0.06
Toolik Lake	16	27-May-13	GF/F	Dark-Control 12 hours	0.91
Toolik Lake	16	27-May-13	0.2 µm	Light-Exposed 12 hours	0.1
Toolik Lake	16	27-May-13	0.2 µm	Dark-Control 12 hours	0.09

Supplementary Figure 1: (Top) van Krevelen diagrams of formulas degraded by bacteria in organic layer (blue squares) and permafrost DOM (red triangles). The marker color intensity scales with molecular weight (Da), shown from 200 to1000. Changes in peak intensities were calculated using the 95% CI of experimental triplicates. Peak intensities decreased by 1 to 50% in organic layer DOM and 1 to 70% in permafrost DOM. Average chemical characteristics are presented in Supplementary Table 1. (Bottom) van Krevelen diagram showing permafrost DOC formulas that were degraded by bacteria and produced by sunlight (red triangles), and organic layer DOC formulas that were degraged by bacteria and sunlight (blue squares).

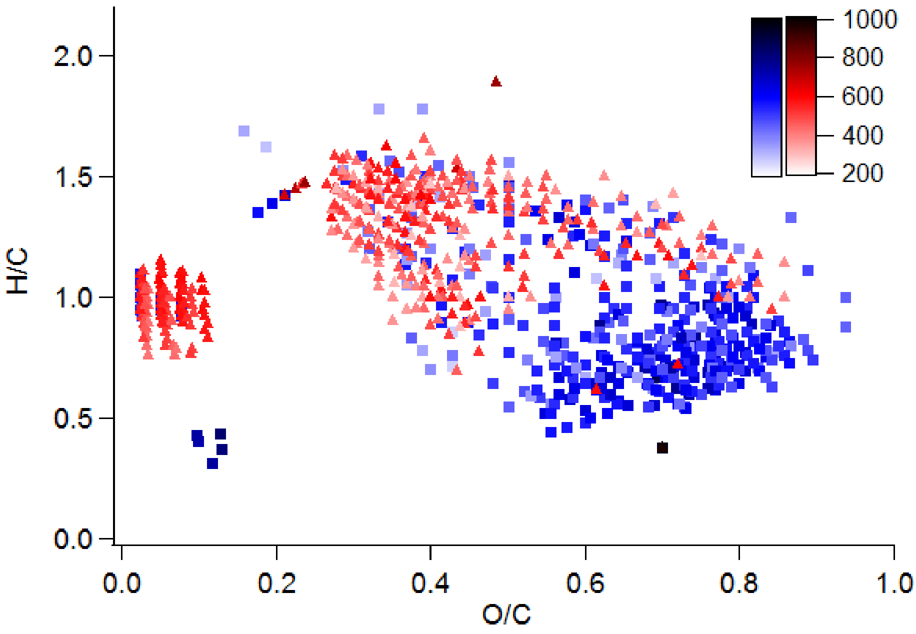

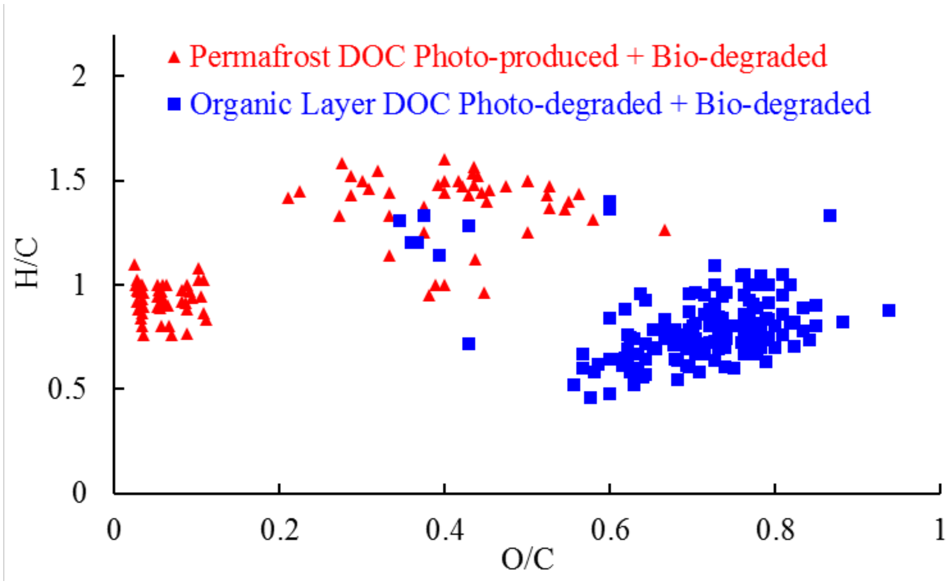

Supplementary Figure 2: (Top) Comparison of spectral shape of simulated sunlight (Atlas XLS+ solar simulator) and natural clear-sky, mid-day June sunlight at Toolik Field Station, AK. (Bottom) Comparison of spectral intensity of simulated sunlight to daily averaged natural sunlight throughout June of 2013. On average, simulated sunlight was 2.4-fold more intense than natural sunlight (range 1.6 to 4.5-fold higher intensity). The simulated sunlight spectrum was acquired using an Ocean Optics USB2000+, while the natural sunlight spectrum was acquired following [7].

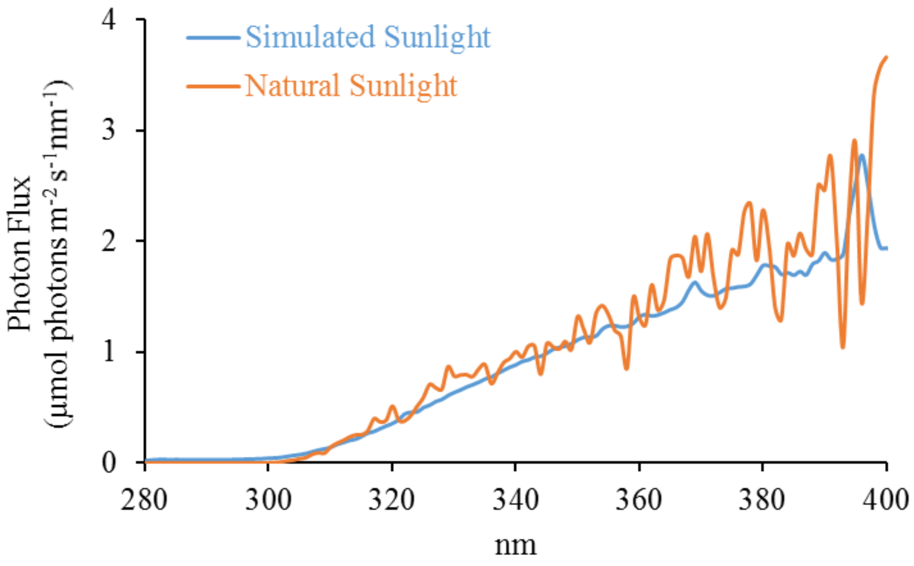

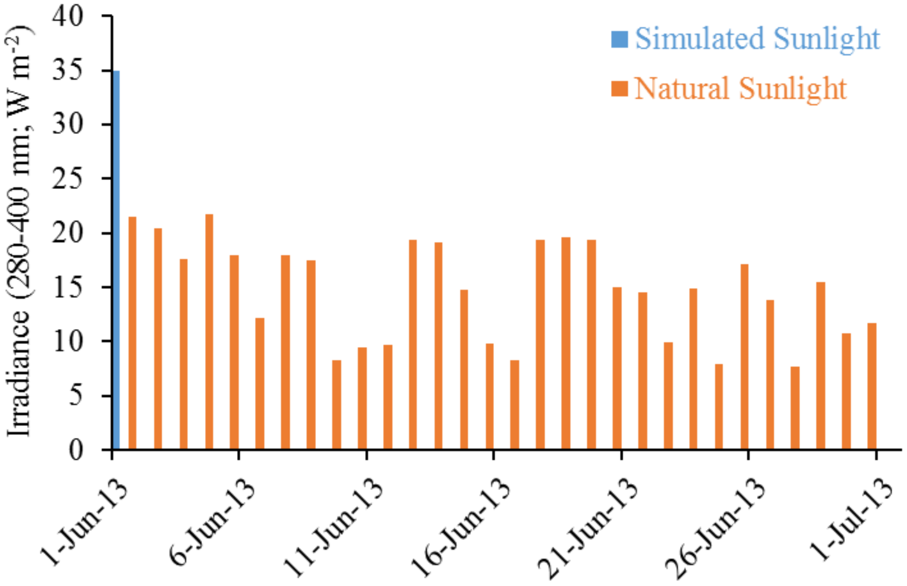

Supplementary Figure 3: Average shifts in compound class distribution for (**A**) photo-degraded and (**B**) photo-produced formulas in DOC leached from the active and permafrost layer of five different soil types on the North Slope of Alaska. Chemical attributes of the soil DOC sources are described in Supplementary Table 3. These results indicate that the changes to DOC chemical composition by sunlight follow a similar pattern across a wide range of Alaskan Arctic soils (i.e., larger, more aromatic, and more oxidized DOC is degraded by sunlight into smaller, more aliphatic, and less oxidized DOC; Supplementary Table 4). It follows that the response of microbes to photochemical changes in DOC chemical composition reported for Imnavait moist acidic tundra DOC in this study (e.g., Figs. 2 and 3 in the main text) likely apply to a wide range of sunlit surface waters draining permafrost soils. Criteria for assigning formulas to each compound class are described in Supplementary Table 3. Error bars indicate ± 1 standard error of the mean.

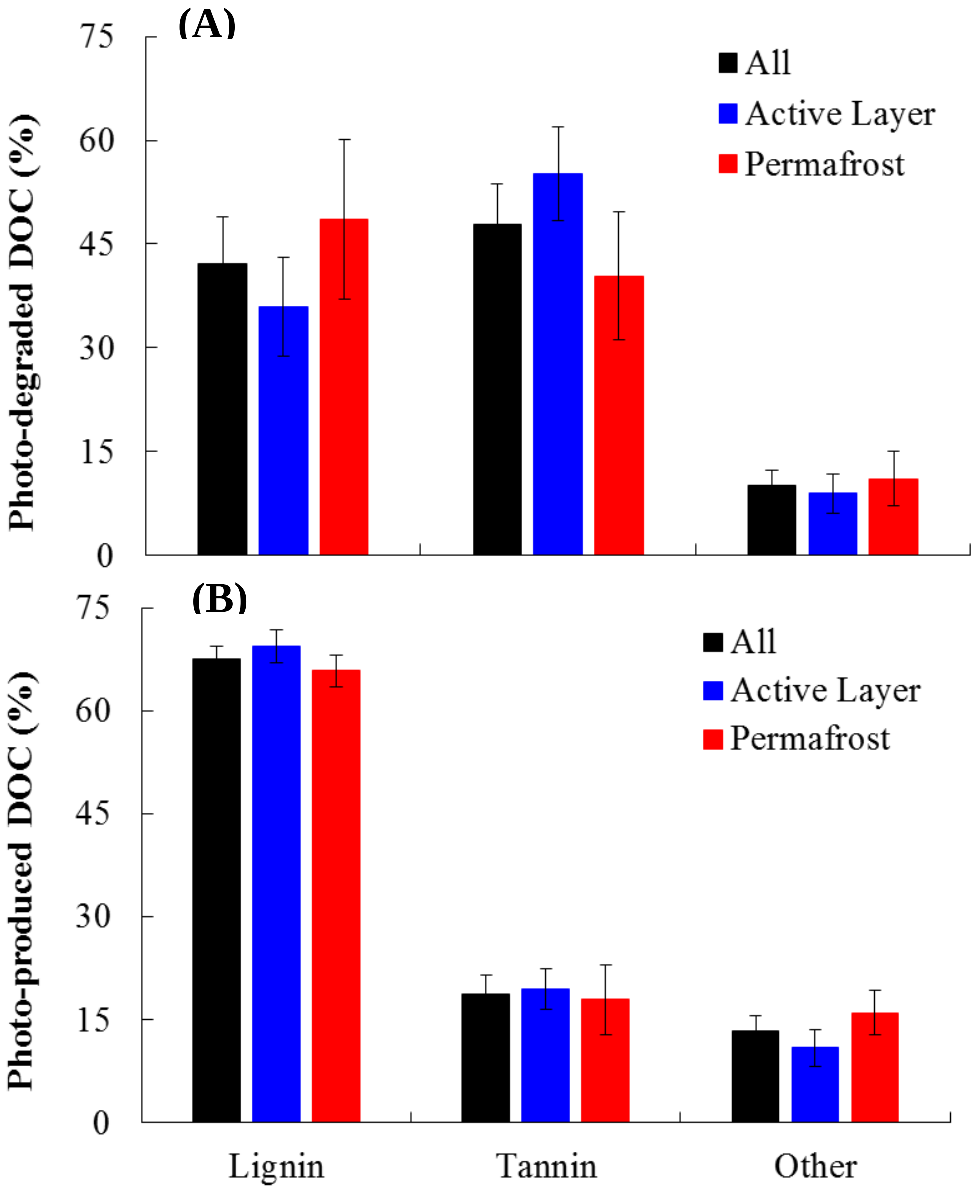

Supplementary Figure 4: Taxonomic information for bacterial communities in each sample are expressed as a fraction of total sequences.

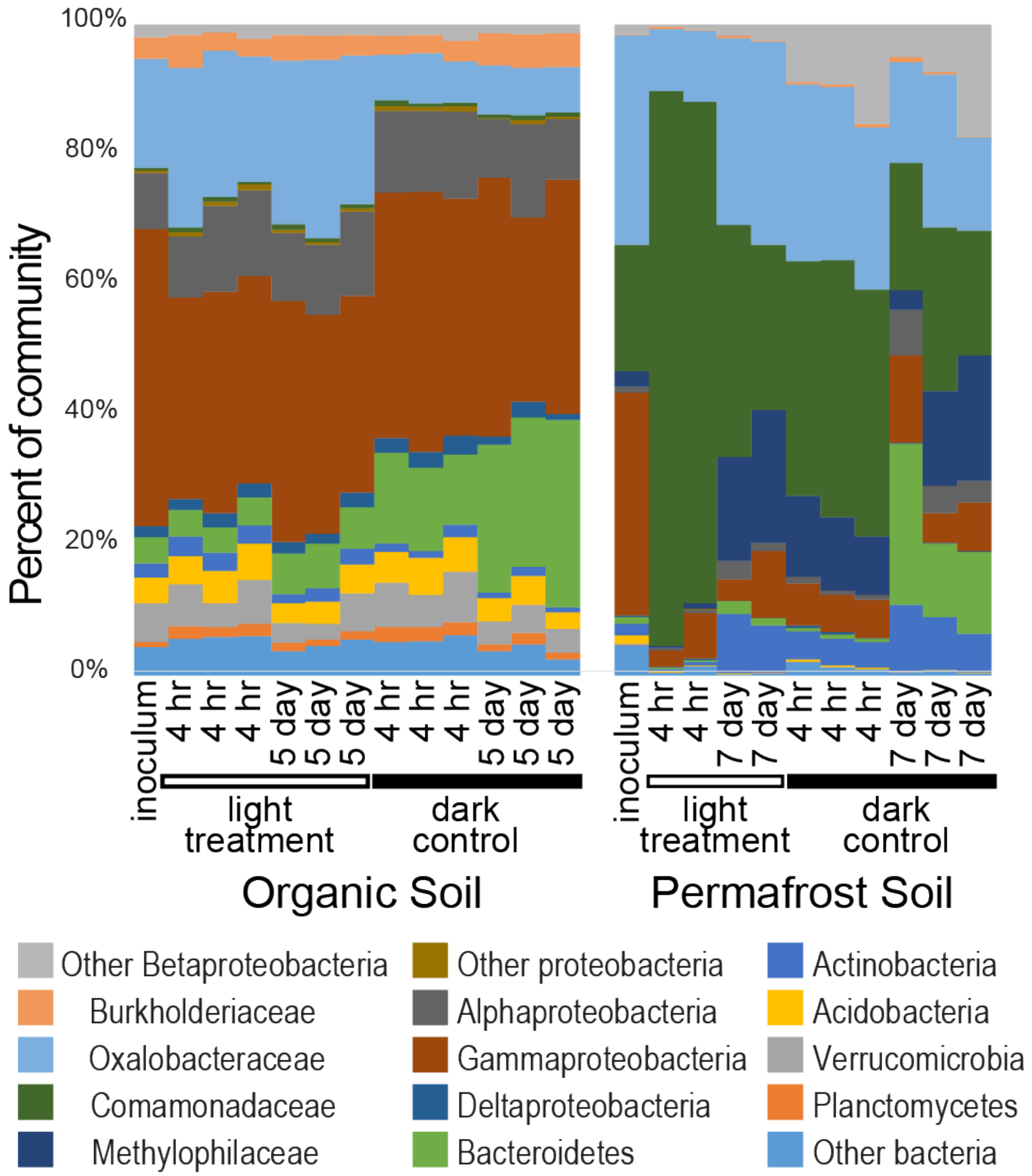

Supplementary References:

1. Ward, C. P. & Cory, R. M. Complete and partial photo-oxidation of dissolved organic matter draining permafrost soils. *Environ. Sci. Technol.* **50,** 3545–3553 (2016).

2. Koch, B. P. & Dittmar, T. From mass to structure: An aromaticity index for high-resolution mass data of natural organic matter. *Rapid Commun. Mass Spectrom.* **30,** 250 (2016).

3. Walker, D. A. *et al.* The Circumpolar Arctic vegetation map. *J. Veg. Sci.* **16,** 267–282 (2005).

4. Kling, G. W., Kipphut, G. W., Miller, M. M. & O'Brien, J. W. Integration of lakes and streams in a landscape perspective: the importance of material processing on spatial patterns and temporal coherence. *Freshw. Biol.* **43,** 477–497 (2000).

5. Weishaar, J. L. *et al.* Evaluation of specific ultraviolet absorbance as an indicator of the chemical composition and reactivity of dissolved organic carbon. *Environ. Sci. Technol.* **37,** 4702–8 (2003).

6. Ward, C. P. & Cory, R. M. Chemical composition of dissolved organic matter draining permafrost soils. *Geochim. Cosmochim. Acta* **167,** 63–79 (2015).

7. Cory, R. M., Ward, C. P., Crump, B. C. & Kling, G. W. Sunlight controls water column processing of carbon in arctic fresh waters. *Science* **345,** 925–928 (2014).

8. Cory, R. M., Crump, B. C., Dobkowski, J. A. & Kling, G. W. Surface exposure to sunlight stimulates CO2 release from permafrost soil carbon in the Arctic. *Proc. Natl. Acad. Sci. U. S. A.* **110,** 3429–3434 (2013).

9. Judd, K. E., Crump, B. C. & Kling, G. W. Bacterial responses in activity and community composition to photo-oxidation of dissolved organic matter from soil and surface waters. *Aquat. Sci.* **69,** 96–107 (2007).

10. Judd, K. E., Crump, B. C. & Kling, G. W. Variation in dissolved organic matter controls bacterial production and community composition. *Ecology* **87,** 2068–79 (2006).

11. Nalven, S. G. Decoding DOM Degradation with Metatranscriptomics : How Do Sunlight and Microbial Communities Interact to Degrade Dissolved Organic Matter in Arctic Freshwaters? (Oregon State University, 2016).

12. Adams, H. E., Crump, B. C. & Kling, G. W. Isolating the effects of storm events on arctic aquatic bacteria: Temperature, nutrients, and community composition as controls on bacterial productivity. *Front. Microbiol.* **6,** 1–13 (2015).

13. Cory, R. M., Harrold, K. H., Neilson, B. T. & Kling, G. W. Controls on DOM degradation in a headwater stream: the influence of DOM amount and lability, light attenuation and exposure. *Biogeosciences* **12,** 6669–6685 (2015).

14. Page, S. E., Logan, J. R., Cory, R. M. & McNeill, K. Evidence for dissolved organic matter as the primary source and sink of photochemically produced hydroxyl radical in arctic surface waters. *Environ. Sci. Process. Impacts* **16,** 807–22 (2014).
